# SNR Enhancement for Comparator-Based Ultra-Low-Sampling Φ-OTDR System Using Compressed Sensing

**DOI:** 10.3390/s24113279

**Published:** 2024-05-21

**Authors:** Zhenyu Xiao, Xiaoming Li, Haofei Zhang, Xueguang Yuan, Yang-An Zhang, Yuan Zhang, Zhengyang Li, Qi Wang, Yongqing Huang

**Affiliations:** 1 State Key Laboratory of Information Photonics and Optical Communications, Beijing University of Posts and Telecommunications, Beijing 100876, China; zyxiao@bupt.edu.cn (Z.X.); zhang@bupt.edu.cn (Y.-A.Z.); zhang_yuan@bupt.edu.cn (Y.Z.); lizhengyang@bupt.edu.cn (Z.L.); wangqi@bupt.edu.cn (Q.W.); yqhuang@bupt.edu.cn (Y.H.); 2 School of Electronic Engineering, Beijing University of Posts and Telecommunications, Beijing 100876, China; 3 No. 208 Research Institute of China Ordnance Industries, Beijing 102202, China; zgbq208@163.com (X.L.); jeamszhf@163.com (H.Z.)

**Keywords:** compressed sensing, phase-sensitive optical time-domain reflectometry, signal-to-noise ratio, ultra-low sampling, data volume

## Abstract

The large amount of sampled data in coherent phase-sensitive optical time-domain reflectometry (Φ-OTDR) brings heavy data transmission, processing, and storage burdens. By using the comparator combined with undersampling, we achieve simultaneous reduction of sampling rate and sampling resolution in hardware, thus greatly decreasing the sampled data volume. But this way will inevitably cause the deterioration of detection signal-to-noise ratio (SNR) due to the quantization noise’s dramatic increase. To address this problem, denoising the demodulated phase signals using compressed sensing, which exploits the sparsity of spectrally sparse vibration, is proposed, thereby effectively enhancing the detection SNR. In experiments, the comparator with a sampling parameter of 62.5 MS/s and 1 bit successfully captures the 80 MHz beat signal, where the sampled data volume per second is only 7.45 MB. Then, when the piezoelectric transducer’s driving voltage is 1 Vpp, 300 mVpp, and 100 mVpp respectively, the SNRs of the reconstructed 200 Hz sinusoidal signals are respectively enhanced by 23.7 dB, 26.1 dB, and 28.7 dB by using compressed sensing. Moreover, multi-frequency vibrations can also be accurately reconstructed with a high SNR. Therefore, the proposed technique can effectively enhance the system’s performance while greatly reducing its hardware burden.

## 1. Introduction

Fiber-optic distributed acoustic sensors (DAS) based on phase-sensitive optical time-domain reflectometry (Φ-OTDR) have been widely applied in many fields such as structural health monitoring [1,2], pipeline monitoring [3,4], and seismic wave detection [5,6] owing to their high sensitivity, fast response, and long sensing distance. Φ-OTDR utilizes the Rayleigh backscattered (RBS) light generated by highly coherent optical pulses along the sensing fiber for distributed vibration sensing. When the external vibration acts on the sensing fiber, the optical path length in the vibration area will change, thus causing variations in the amplitude and phase of the RBS lightwave [7]. Since the RBS amplitude variation is not linearly related to the vibration-induced dynamic strain, the RBS phase information is used to realize linear extraction of the vibration waveform in Φ-OTDR [8].

Among various phase demodulation schemes of Φ-OTDR [9,10,11,12,13,14,15], coherent detection is frequently employed due to its high signal-to-noise ratio (SNR) and simple configuration [12,13,14,15]. However, in the coherent Φ-OTDR system, the central frequency of the beat signal is usually comparatively high, such as the common 80~200 MHz [16,17,18], which requires a data acquisition card (DAQ) with a high sampling rate for signal sampling according to the Nyquist sampling theorem. In addition, to ensure the quantization accuracy of sampled data, the sampling resolution of current DAQs is basically 8 bits and above [18,19,20]. As a result, the required high-performance DAQ configuration will greatly increase the cost of the sensing system. More importantly, a large amount of raw data sampled by the DAQ will bring heavy data transmission, processing, and storage burdens to the system, especially in the long-term or long-range monitoring scenarios [21,22], which greatly limits the real-time vibration monitoring performance of DAS.

To reduce the burdens brought by a large amount of sampled data in the coherent Φ-OTDR system, some workable methods have been proposed [23,24,25,26]. One method is to extract only the specific phase information at a sampling rate corresponding to the system’s spatial resolution [23]. Although this method reduces the data volume to a certain extent, it is not suitable for scenarios which require dense sampling points along the distance axis, such as seismic wave monitoring. In 2019, the undersampling method was introduced to decrease the high sampling rate requirement in coherent Φ-OTDR [24]. The off-line experimental result showed that the beat signal with a 200 MHz central frequency could be successfully collected with a sub-Nyquist rate as low as 71 MS/s, thus reducing the sampled data volume. In 2022, An ultra-low sampling resolution technique was proposed to solve the data storage problem in heterodyne Φ-OTDR [25]. The results showed that the vibration-induced optical phase variation could be correctly demodulated from the 1-bit-resolution data, thereby effectively reducing the stored data volume.

Then, a combination scheme using undersampling and ultra-low sampling resolution was proposed to solve the data storage problem with a high space saving ratio [26]. However, the feasibility of the combination technique has only been preliminarily demonstrated in the digital domain, and its performance in the practical sensing system remains to be investigated, especially in real-time application scenarios. Moreover, a reduction in the sampling rate or sampling resolution of DAQ will cause an increase in quantization noise within the frequency band of interest, thereby reducing the SNR of the demodulated phase signals [26,27,28]. When both are reduced at the same time to greatly reduce the system’s sampled data volume, the SNR will even seriously deteriorate, thus affecting the vibration detection capability of the Φ-OTDR system.

In this paper, we introduce the compressed sensing technique for the comparator-based ultra-low-sampling Φ-OTDR system to effectively enhance the SNR of the demodulated phase signals while greatly reducing the sampled data volume. Firstly, a comparator combined with undersampling, instead of the common high-performance DAQ, is used to significantly reduce the sampled data volume in hardware. Then, for the spectrally sparse vibration signals, the application of compressed sensing effectively solves the SNR deterioration problem caused by the dramatic increase in quantization noise in the ultra-low-sampling Φ-OTDR system, thus enhancing the detection SNR. Meanwhile, the compression and reconstruction process of the phase signals based on compressed sensing is given and analyzed. Finally, a series of experiments are conducted to demonstrate the effectiveness of the proposed technique, which has great application potential in improving the system’s performance while reducing its hardware burden.

## 2. Principles and Theoretical Analysis

### 2.1. Principle of Comparator-Based Ultra-Low-Sampling Φ-OTDR System

In the coherent Φ-OTDR system, the one-dimensional (1-D) backscattering model is commonly used to describe the Rayleigh backscattering in the optical fiber. When an optical probe pulse with a width of $T_{p}$ is injected into the sensing fiber, the generated RBS light will be mixed with the local oscillator (LO) light to produce a beat light. After photoelectric conversion, the beat light will be converted into an electric beat signal, of which the alternating current (AC) component can be described as [29]:

(1) $$\begin{array}{l} I(t)=2E_{0}E_{LO}\sum_{i=1}^{N}a_{i}\exp(-\alpha \nu \tau_{i})\\ \;\;\;\;\;\;\;\;\;\;\cdot \cos[2\pi \Delta ft-2\pi (f_{0}+\Delta f)\tau_{i}+\varphi (\tau_{i})]\mathrm{rect}\left[\left(t-\tau_{i}\right)/T_{p}\right] \end{array}$$

where $E_{0}$ and $E_{LO}$ are the electric field intensity of the incident light and LO light, respectively; $a_{i}$, $\tau_{i}$, and $\varphi (\tau_{i})$ are the amplitude, relative delay, and random phase of the *i*-th scattering unit, respectively; *N* is the total number of scattering units; *α* is the fiber attenuation coefficient; *ν* is the velocity of light in the sensing fiber; Δf is the frequency shift introduced by the acousto-optic modulator (AOM); and rect(·) represents the rectangular pulse function. According to the Wiener–Khinchin theorem, the instantaneous power spectrum of I(t) can be obtained through the Fourier transform of the autocorrelation function of Equation (1) [30]:

(2) $$S(t,f)≃E_{0}^{2}E_{LO}^{2}T_{p}^{2}\bar{a^{2}}\exp(-2\alpha \nu t){\sin c}^{2}[(f\pm \Delta f)T_{p}]$$

where sinc(x)=sin(πx)/πx. From Equation (2), we can know that the central frequency and approximate full width at half-maximum (FWHM) of the beat signal’s power spectrum are Δf and $0.89/T_{p}$. Thus, combined with the common parameter settings of the coherent Φ-OTDR system, the beat signal can be regarded as a bandpass signal after simple bandpass filtering.

Since the beat signal is bandpass, the undersampling technique can be used to reduce the required sampling rate in the coherent Φ-OTDR system. The beat signal can be acquired at a lower sampling rate while avoiding spectrum aliasing and the sampling rate $f_{s}$ only needs to satisfy [24]:

(3) $$\frac{2\Delta f+B}{m}\leq f_{s}\leq \frac{2\Delta f-B}{m-1}$$

where *m* is an integer with a range of 1≤m≤(2Δf+B)/2B; *B* is the bandwidth of the bandpass filter (BPF) following the photodetector. *B* should be not less than the FWHM of the beat signal’s spectrum which is related to the optical pulse’s width, i.e., $B\geq 0.89/T_{p}$. Thus, combined with the parameter settings in our experiments (Δf = 80 MHz, $T_{p}$ = 100 ns), while we take *B* to be 15 MHz. For the selection of *m*, we need to consider the system’s feasible sampling rate and its tolerance to the noise [28]. As *m* increases, the feasible sampling rate value will become smaller, but at the same time, the value range will narrow accordingly which means that the restriction on the sampling rate value will become greater, and the SNR of the sampled signals will also become worse. In experiments, we choose $f_{s}$ = 62.5 MS/s (corresponding to *m* = 3) for undersampling, where we can see that the sampling rate is even lower than ∆*f*. Therefore, the undersampling technique can be used to reduce the number of samples collected by DAQ per second.

Another way to reduce the sampled date volume is to decrease the sampling resolution of DAQ. In the current Φ-OTDR systems, the DAQ sampling resolution is basically no lower than 8 bits and some of them even reach 16 bits [26] for higher sampling accuracy. In fact, we are mainly concerned with the phase variation of the beat signal, which is linearly related to the external vibration along the fiber, so the sufficient decrease in its amplitude accuracy to reduce the space occupied by each sample is acceptable. The research shows that, when retaining the main spectral characteristics of the beat signal, constantly reducing the bit number of DAQ, even as low as 1 bit will lead to a continuous decline in the amplitude accuracy of the sampled signal, while the phase demodulation result is still considerable [25]. Therefore, it is feasible to reduce the sampled data volume by reducing the DAQ’s sampling resolution while successfully demodulating the phase information of the beat signal.

Thus, according to the above theory, by using a 1-bit comparator combined with the undersampling technique instead of the DAQ with high sampling performance, we can achieve simultaneous reduction of the required sampling rate and sampling resolution in hardware. This greatly decreases the sampled data volume and mitigates the system’s hardware burden. Meanwhile, it also greatly saves the cost of data acquisition device, which has certain economic advantages. However, due to the simultaneous decrease in sampling rate and sampling resolution of the data acquisition device, the SNR deterioration problem caused by the dramatic increase in quantization noise needs urgent attention [26,27,28]. Especially when the intensity of vibration signals is not large, the deterioration of detection SNR will become noticeable, which will greatly affect the vibration detection capability of the Φ-OTDR system.

### 2.2. Theory of Phase Reconstruction Using Compressed Sensing

To solve the SNR deterioration problem in the ultra-low-sampling Φ-OTDR system, denoising the phase signals based on compressed sensing is a good solution [31,32]. At the signal processing end, the quantization noise can be reduced by compressing and then reconstructing the time-domain phase signal $\varphi (z_{0},t)$ at position $z_{0}$ by taking advantage of the sparsity of the signal.

Since the phase signal $\varphi (z_{0},t)$ is an N×1 dimensional real-valued discrete time-domain signal, it can be represented with the weighting coefficients ${\left\{s_{i}\right\}}_{i=1}^{N}$ on a certain set of N×1 dimensional orthogonal basis ${\left\{\psi_{i}\right\}}_{i=1}^{N}$. Then, $\varphi (z_{0},t)$ is represented as:

(4) $$\varphi \left(z_{0},t\right)=s_{1}\psi_{1}+s_{2}\psi_{2}+\cdots +s_{N}\psi_{N}=\sum_{i=1}^{N}s_{i}\psi_{i}=\Psi s$$

where $\Psi =[\psi_{1},\psi_{2},\cdots ,\psi_{N}]$ is an N×N dimensional matrix and $s={\left[s_{1},s_{2},\cdots ,s_{N}\right]}^{T}$ is an N×1 dimensional coefficient vector. The signal is considered *K*-sparse in the **Ψ** domain if only *K* (K≪N) coefficients are non-zero in ***s***, where *K* is the signal’s sparsity level. Accordingly, **Ψ** and ***s*** are called the sparse transform matrix and the sparse coefficient, respectively. The common sparse transform matrices include discrete Fourier transform (DFT), discrete cosine transform (DCT), and discrete wavelet transform (DWT). It can be seen that $\varphi (z_{0},t)$ and ***s*** are equivalent expressions of the same signal in different transform domains, where the former is based on the time domain and the latter is in the **Ψ** domain.

**Figure 1:**
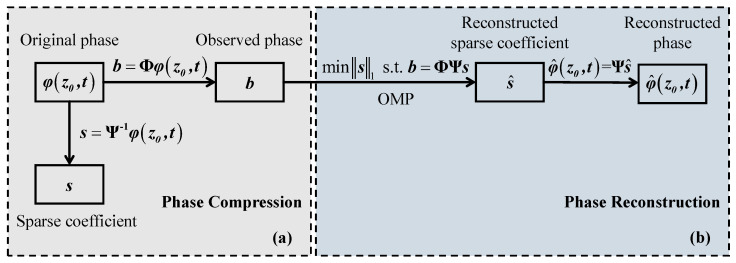
(**a**) The compression and (**b**) reconstruction process of the phase signal based on compressed sensing.

Next, under the condition that the signal $\varphi (z_{0},t)$ is sparse in the **Ψ** domain, an M×N(M≪N) observation matrix **Φ** is used to project the high-dimensional $\varphi (z_{0},t)$ to a low-dimensional space, thereby achieving the signal’s compressed observation. The process is as follows:

(5) $$b=\Phi \varphi \left(z_{0},t\right)=\Phi \Psi s=\Theta s$$

where Θ=ΦΨ represents the sensing matrix with the size of M×N and ***b*** is the M×1 dimensional observation vector, i.e., the compressed signal. Then, a sufficient condition for accurately reconstructing $\varphi (z_{0},t)$ from ***b*** is that **Θ** satisfies the Restricted Isometry Property (RIP) [33], and an equivalent condition is that **Φ** and **Ψ** are incoherent [34]. The compression process of the phase signal based on compressed sensing is shown in Figure 1a.

Given that the frequency spectrum is a key parameter for us to analyze the characteristics of external vibrations and the actual vibration signals are usually band-limited [31], we choose the frequency domain as the sparse domain, that is, the sparse transform matrix is the inverse DFT matrix here. Then, under the premise that the original phase $\varphi (z_{0},t)$ is sparse in the frequency domain, we realize the compressed observation of $\varphi (z_{0},t)$ through the observation matrix **Φ** according to Equation (5), and obtain the observed phase ***b***. Here the random Gaussian matrix is used as **Φ**, which is commonly used in compressed sensing and is incoherent with the inverse DFT matrix.

Finally, we need to reconstruct the original phase based on the known observed phase. Since *M* is much smaller than *N*, we are unable to recover $\varphi (z_{0},t)$ from ***b*** directly. But the sparsity condition of the original signal plays an important role at this moment. Due to the condition that $\varphi (z_{0},t)$ is sparse in the frequency domain, the reconstruction of the sparse set of significant coefficients ***s*** can be achieved by solving the *l*_0_-norm optimization problem:

(6) $$\min{\left\|s\right\|}_{0}\;\;\;s.t.\;\;\;b=\Phi \Psi s$$

while solving the *l*_0_-norm optimization problem is NP hard, which is then transformed into the solution of the *l*_1_-norm optimization problem [33]:

(7) $$\min{\left\|s\right\|}_{1}\;\;\;s.t.\;\;\;b=\Phi \Psi s$$

By applying the orthogonal matching pursuit (OMP) algorithm [35], which is one of the greedy algorithms based on dynamic programming, we can gain the reconstructed value of the sparse coefficient, $\hat{s}$. Then, based on the transform $\hat{\varphi }(z_{0},t)=\Psi \hat{s}$, the reconstructed value of the original phase, $\hat{\varphi }(z_{0},t)$, can finally be obtained. The reconstruction process of the phase signal based on compressed sensing is shown in Figure 1b.

Due to the sparsity of the original phase in the frequency domain, the observed phase retains the global characteristics of the original phase during the compression process, so the subsequent application of relevant algorithms, such as OMP, can reconstruct the original signal with a high probability. While the quantization noise is not sparse in the frequency domain, a considerable part of the noise information is abandoned during the compression process. In this way, the quantization noise is suppressed, and the SNR of the demodulated phase signal is effectively enhanced.

## 3. Experimental Setup and Results

### 3.1. Experimental Setup

**Figure 2:**
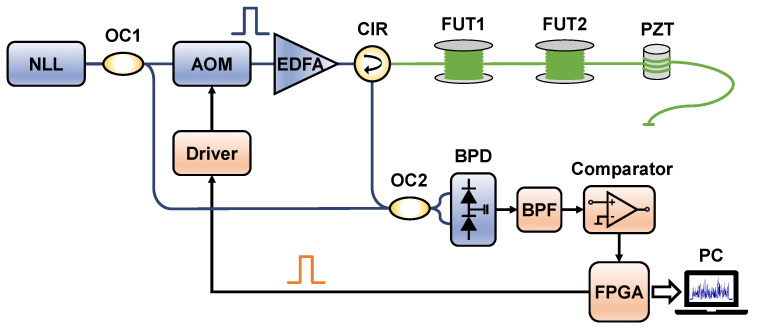
The experimental setup. NLL: narrow linewidth laser; OC: optical coupler; AOM: acousto-optic modulator; EDFA: erbium-doped fiber amplifier; CIR: optical circulator; FUT: fiber under test; PZT: piezoelectric transducer; BPD: balanced photodetector; BPF: bandpass filter; FPGA: field-programmable gate array; PC: personal computer.

The experimental setup of the coherent Φ-OTDR system based on the comparator is shown in Figure 2. A narrow linewidth laser (NLL) working at 1550.12 nm with a 3 kHz linewidth is used as the light source, from which the continuous light is divided into two beams by a 90:10 optical coupler (OC1). One beam (90%) is used as the probe light and the other (10%) acts as the LO light for coherent detection. Then, the probe light is periodically modulated into optical pulses with a repetition rate of 20 kHz and a width of 100 ns by an AOM, which brings in an 80 MHz frequency shift. After being amplified by an erbium-doped fiber amplifier (EDFA), the optical probe pulses are then launched into the 4.52 km fiber under test (FUT) through an optical circulator (CIR). A piezoelectric transducer (PZT) with about 10 m long fiber wound is placed at the end of the FUT, at approximately 4.5 km, as a vibration source. Next, the RBS light returned from the FUT beats with the LO light at a 50:50 optical coupler (OC2) and is then detected by a balanced photodetector (BPD) with a 200 MHz bandwidth. The electrical beat signal from the BPD passes through a BPF, of which the central frequency and bandwidth are 80 MHz and 15 MHz, respectively. Lastly, the bandpass signal is sampled by a comparator (TI TLV3501) and then processed in real-time by a field-programmable gate array (FPGA). The vibration detection results are displayed on a computer.

It is worth noting that the probe pulses used to drive the AOM are generated by the FPGA. Since the pulse width is 100 ns in our system, which means that the FWHM of the beat signal’s power spectrum is ~8.9 MHz, the BPF with a central frequency of 80 MHz and a bandwidth of 15 MHz is selected. Through the corresponding clock configuration of the FPGA, the equivalent sampling rate of the comparator is set to 62.5 MS/s (*m* = 3) for undersampling. Moreover, the threshold voltage of the comparator is configured as a fixed zero level. In this way, the comparator can accurately obtain the 1-bit sign information of the beat signal so that its main frequency characteristics can be preserved.

### 3.2. Results of Comparator-Based Ultra-Low-Sampling Φ-OTDR System

**Figure 3:**
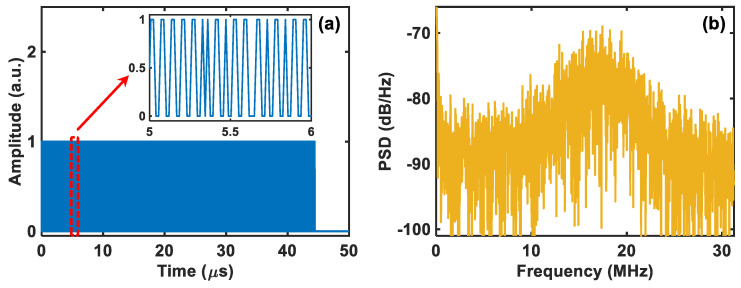
(**a**) The beat signal’s trace of one complete period acquired by the comparator and (**b**) the corresponding power spectrum density (PSD).

To evaluate the practical performance of the comparator-based ultra-low-sampling Φ-OTDR system, several experiments are carried out successively. Firstly, without applying a driving signal on the PZT, we conduct a preliminary analysis on the result acquired by the comparator. The acquired beat signal’s trace of one complete period is depicted in Figure 3a, where the partial enlargement between 5~6 s shows the trace details. It can be clearly seen that the amplitude value only has two states of 0 and 1, that is, the resolution of the sampled signal is 1 bit. Figure 3b shows the corresponding power spectrum density (PSD), from which we can obtain that its central frequency is approximately 17.5 MHz. The result is completely consistent with that of undersampling the 80 MHz beat signal at a 62.5 MS/s sampling rate [24], and more importantly, the main spectral characteristics of the beat signal are well preserved. Thus, it is feasible to use the 1-bit-resolution beat signal sampled by the comparator for phase demodulation.

**Figure 4:**
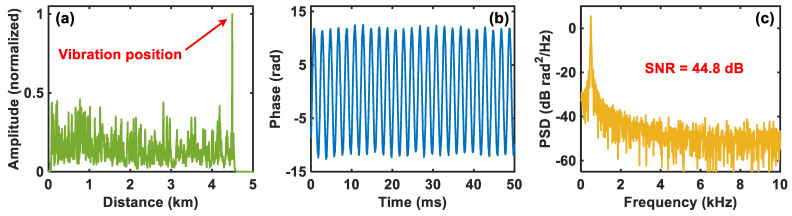
Detection results of the 500 Hz and 5 Vpp (peak-to-peak voltage) sinusoidal vibration signal of the comparator-based ultra-low-sampling Φ-OTDR system. (**a**) Vibration location result. (**b**) Demodulated vibration waveform from the PZT and (**c**) the corresponding PSD.

Then, a 500 Hz and 5 Vpp (peak-to-peak voltage) sinusoidal driving signal is applied on the PZT to simulate the external vibration. By using the moving differential method, the vibration location result based on the amplitude information is shown in Figure 4a. It can be clearly determined that the vibration signal is located at 4502 m, which is consistent with where the PZT is placed. After phase demodulation, the demodulated vibration waveform at 4502 m and the corresponding PSD are depicted in Figure 4b,c, respectively, from which we can see that the demodulated vibration signal is exactly the 500 Hz sinusoidal signal and its SNR is about 44.8 dB. The above results show that the comparator-based ultra-low-sampling Φ-OTDR system can accurately detect the vibration signal.

In terms of reducing the sampled data volume, for the beat signal whose central frequency is 80 MHz, the typical and commonly used sampling parameter is 250 MS/s and 14 bits [18,36,37], while the sampling parameter of the comparator used in our system is 62.5 MS/s and 1 bit. In this way, the data volume per second of the sampled signal is reduced from 417.23 MB to 7.45 MB, where the latter is only ~1.79% of the former (i.e., a reduction of up to 56 times), showing the efficiency of the ultra-low-sampling system in reducing the sampled data volume.

**Figure 5:**
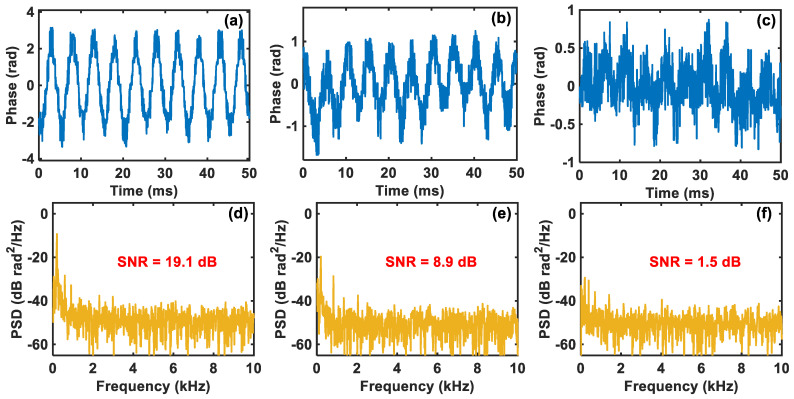
Demodulated vibration waveforms when the driving voltage is (**a**) 1 Vpp, (**b**) 300 mVpp, and (**c**) 100 mVpp, respectively. The corresponding PSDs of the demodulated vibration waveforms when the driving voltage is (**d**) 1 Vpp, (**e**) 300 mVpp, and (**f**) 100 mVpp, respectively.

However, ultra-low sampling rate and sampling resolution will inevitably cause an increase in the system’s quantization noise. Especially when the vibration signal’s intensity is not large, the detection SNR will be seriously deteriorated. Thus, we continuously decreased the applied vibration intensity to observe the performance of the demodulated vibration signal. The PZT is driven by 200 Hz sinusoidal signals with amplitudes of 1 Vpp, 300b mVpp, and 100 mVpp, respectively. Figure 5a–c show the corresponding demodulated vibration waveforms. It is obvious that as the amplitude of the driving signal decreases, the demodulated waveform becomes increasingly rough and distorted. Especially when the driving voltage is 100 mVpp, the demodulated vibration waveform has been severely distorted, where we can hardly distinguish the sinusoidal waveform. The PSDs of these demodulated waveforms are plotted in Figure 5d–f, respectively, where we can obtain that the corresponding SNRs are 19.1 dB, 8.9 dB, and 1.5 dB. The serious deterioration of the detection SNR indicates that the comparator-based ultra-low-sampling Φ-OTDR system has a poor performance in detecting small vibration signals due to the influence of increased quantization noise.

### 3.3. Analysis of Compressed Sensing Results

**Figure 6:**
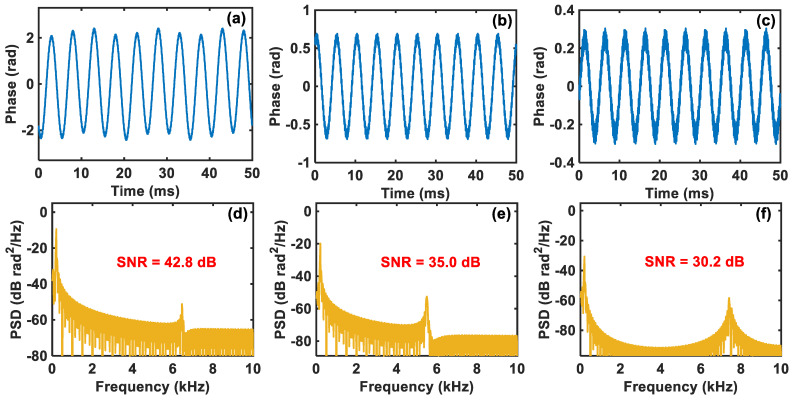
Reconstructed vibration waveforms using compressed sensing when the driving voltage is (**a**) 1 Vpp, (**b**) 300 mVpp, and (**c**) 100 mVpp, respectively. The corresponding PSDs of the reconstructed vibration waveforms when the driving voltage is (**d**) 1 Vpp, (**e**) 300 mVpp, and (**f**) 100 mVpp, respectively.

Through a sequence of experimental tests, 30% of the demodulated data is used for the vibration signal’s compression and further reconstruction, which means that the value of *M* is 30% of *N*. Then, the demodulated vibration signals in Figure 5 are compressed and reconstructed according to the processing flow of compressed sensing shown in Figure 1. The reconstructed vibration waveforms when the driving voltage is 1 Vpp, 300 mVpp, and 100 mVpp, respectively, are given in Figure 6a–c. Compared with the original demodulated waveforms shown in Figure 5a–c, the reconstructed vibration waveforms after compressed sensing look significantly smoother, and the distortions present in the original demodulated waveforms are also well compensated, especially when the driving voltage is 100 mVpp.

The corresponding PSDs of the reconstructed vibration waveforms are plotted in Figure 6d–f, respectively, from which we can know that their SNRs are 42.8 dB, 35.0 dB, and 30.2 dB, respectively. Compared with the original results shown in Figure 5d–f, the noise floors in Figure 6d–f are obviously lowered, indicating that the disruptive noise is suppressed, and the SNRs are significantly improved by 23.7 dB, 26.1 dB, and 28.7 dB, respectively. Therefore, the compressed sensing technique can effectively enhance the SNR of phase demodulation in the comparator-based ultra-low-sampling Φ-OTDR system, thereby greatly improving the system’s detection ability to detect small vibration signals. It is worth mentioning that the PSDs in Figure 6d–f have different second peak interferences, which are introduced during the compression and reconstruction process of the vibration signals and are related to the vibration signals’ SNR, the parameters of the reconstruction algorithm, etc.

**Figure 7:**
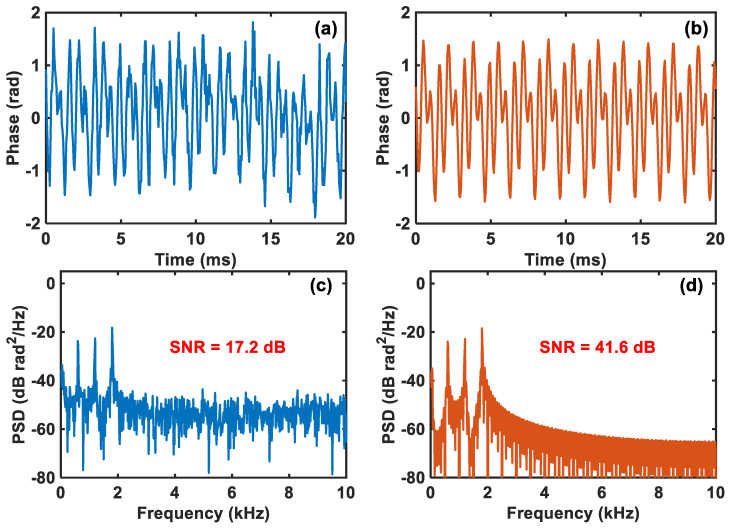
(**a**) Original demodulated vibration waveform and (**b**) reconstructed vibration waveform using compressed sensing. The corresponding PSDs of (**c**) the original demodulated vibration waveform and (**d**) the reconstructed vibration waveform.

To investigate the noise suppression capability of compressed sensing in the comparator-based ultra-low-sampling Φ-OTDR system for the multi-frequency vibration, the PZT is driven by an 800 mVpp multi-frequency vibration signal with three frequency components, 600 Hz, 1.2 kHz, and 1.8 kHz. The original demodulated vibration waveform and the reconstructed one with compressed sensing are plotted in Figure 7a,b, respectively, where we can see that the reconstructed waveform is obviously smoother than the original waveform and it even seems to be almost undisturbed by noise. Then, for further analysis from the spectrum, the corresponding PSDs of both are respectively shown in Figure 7c,d, from which it is clearly seen that all three frequency components are accurately detected by both and the reconstructed signal has a significantly lower noise floor than the original signal. Correspondingly, the SNRs of the two in Figure 7c,d are 17.2 dB and 41.6 dB, respectively, indicating an SNR enhancement of 24.4 dB. Therefore, the noise suppression capability of compressed sensing in the system for multi-frequency vibration events is also considerable.

## 4. Conclusions

In this work, the compressed sensing technique was introduced for the comparator-based ultra-low-sampling Φ-OTDR system to reduce the sampled data volume with a high detection SNR. By using the comparator combined with undersampling, the sampled data volume was greatly decreased in hardware. Then, the application of compressed sensing solved the SNR deterioration problem caused by the dramatic increase in quantization noise in the ultra-low-sampling system, thus effectively improving the SNR. While the premise for compressed sensing to work is that the vibration signals must be spectrally sparse, which is usually satisfied in some scenarios. In experiments, the comparator with a parameter of 62.5 MS/s and 1 bit successfully captured the 80 MHz beat signal, where the sampled date volume is only 7.45 MB/s. For the 200 Hz sinusoidal vibration, when the driving voltage was 1 Vpp, 300 mVpp, and 100 mVpp, respectively, the SNRs of the reconstructed signals were respectively enhanced by 23.7 dB, 26.1 dB, and 28.7 dB with compressed sensing. Therefore, the proposed technique has great application potential in reducing the system’s hardware burden with a good detection performance.

## Data Availability

The data presented in this study are available upon request from the corresponding author. The data are not publicly available due to privacy restrictions.
